# Characterization of a Potent New-Generation Antimicrobial Peptide of *Bacillus*

**DOI:** 10.3389/fmicb.2021.710741

**Published:** 2021-08-24

**Authors:** Shilja Choyam, Priyanshi M. Jain, Rajagopal Kammara

**Affiliations:** Department of Protein Chemistry and Technology, Faculty of AcSIR, CSIR-CFTRI, Mysore, India

**Keywords:** bacteriocins, *Bacillus* antimicrobial peptides, GRAS organisms, probiotics, *Listeria* monocytogenes, *Vibrio harveyi*

## Abstract

An antimicrobial peptide [*Bacillus* antimicrobial peptide (BAMP)] produced by *Bacillus paralicheniformis* was isolated from the Indian traditional fermented food and characterized. The antimicrobial peptide BAMP showed many unique features such as thermostability (4.0–125°C), pH tolerance (pH 2.0–9.0), and resistance to physiological enzymes (trypsin, chymotrypsin, pepsin, proteinase K, protease, and catalase), and food-grade metal salts do not inhibit the activity. The broad spectrum of BAMP (antimicrobial activity) makes it a suitable candidate for food preservation as well as antimicrobial therapy. BAMP was found to exhibit a bacteriostatic effect on *Salmonella typhi* and controls the viability of *Listeria monocytogenes* in chicken meat efficiently. BAMP was found to establish eubiosis, as it is not antagonistic to *Lactobacillus*. Its non-hemolytic nature makes it suitable for therapy. Various genome prediction tools were adopted and applied to understand their localization, gene arrangement, and type of antimicrobials. Founded on its superior functional attributes, BAMP is a potent new-generation antimicrobial peptide.

## Introduction

A few bacteria produce low-molecular-weight antimicrobials known as Ocins. Bacteriocins are identified as ribosomally synthesized (RPS) antimicrobial peptides. Bacitracin is a non-ribosomal (NRPS) synthesized peptide, usually synthesized and secreted by *Bacillus* spp. The characteristics of various bacteriocins such as source, features, and use in the food industry are studied/reported (Choyam et al., 2019). During the process of making various foods for long durability, the food industry incorporates chemical additives that elicit toxic elements. The introduction of chemical-based food additives also brings a change of taste and flavor and lowers the quality of food, further causing a health hazard. Therefore, natural food additives/preservatives such as bacteriocins and bacitracin may play an essential role in the maintenance of food taste, color, and flavor in the long term. Thus, natural antimicrobials such as bacitracin and bacteriocins from probiotic or generally recognized as safe (GRAS) microorganisms promise safety in use as a preservative in meat, aquatic, vegetable, and dairy industries (Stevens et al., 1991; Cleveland et al., 2001; Deegan et al., 2006; Settanni and Corsetti, 2008).

There are four different classes of bacteriocins, namely, I, II, III, and IV. The class I bacteriocins are usually produced by LAB; their molecular weight ranges from 1 to 5 kDa (Boman, 1995), and posttranslational modifications are essential for their function. They are known as lantibiotics, containing lanthionine, an unusual amino acid (Nissen and Nes, 1997). A few examples of class I are Nisin, Subtylin, and Variacin 8 (Gross and Morell, 1971; Gross and Kiltz, 1973; Gilmore et al., 1994). Class II bacteriocins are thermostable, and posttranslational modifications are not required and do not contain the lanthionines hence they are non-lantibiotics. Class II bacteriocins are further divided into three subclasses, namely, IIa, IIb, and IIc (Ennahar et al., 2000). A few well-known class II types are pediocin PA-1, lactacin F, and enterocin AS-48 (Nissen et al., 2009). The class III bacteriocins are thermolabile and larger than 10 kDa (Savadogo et al., 2006). They are subclassified as class IIIa or bacteriolysins and class IIIb or non-lytic bacteriocins. Lysostaphin is the best example (Bastos et al., 2010). Plantaricin S and leuconocin S belong to class IV bacteriocins; they are complex proteins containing lipid or carbohydrate moieties (Upreti and Hinsdill, 1975; Oman et al., 2011). Recently, Cotter et al. (2005) introduced a universal method of bacteriocin classification.

The most widely (commercially) used bacteriocins derived from *Lactobacillus* are Nisin, Pediocin (Broughton, 1990; Twomey et al., 2002), and antimicrobial peptides like bacitracin from *Bacillus* spp. However, the ability of Nisin to function in the presence of food preservatives is yet to be known. Besides, Nisin is thermolabile and protease sensitive, with a narrow (pH range 7.0–8.0) spectrum of activity and specifically, it does not work against *Salmonella*. Therefore, a few limitations still exist for its use in the food industry and human therapy. Thus, the present need is to discover potent and indigenous antimicrobials either bacteriocins or bacitracin. The other well-known antimicrobial peptide is bacitracin, which is a mixture of 10 cyclic dodecapeptides, non-ribosomally synthesized by *B. subtilis* and *B. paralicheniformis* (Azevedo et al., 1993; Ishihara et al., 2002). It is well investigated that bacitracin is primarily active against Gram-positive bacteria only (Rietkötter et al., 2008). Bacitracin is of different types with varying degrees of antibacterial activity. They include bacitracin A1, A2, B1, B2, B3, C, D1, D2, E, F, H1, H2, H3, I1, I2, and I3 with few amino acid differences.

The primary aim of the present manuscript lies in characterizing bacitracin derived from *B. paralicheniformis*, finally concluding their importance in the food industry. Based on its characteristics and functional attributes, we reason that it is a potent antimicrobial agent.

## Materials and Methods

### Bacterial Strains Used in the Study

All the microorganisms, including indicator strains, were received from the Microbial Type Culture Collection (MTCC-IMT, India), and they were grown in Luria-Bertani (LB), De Man, Rogosa, and Sharpe (MRS), brain heart infusion (BHI) media (Hi-media), or nutrient broth (NB). *B. paralicheniformis*, a novel isolate showing antimicrobial activity (isolated from Himachal Pradesh traditional foods), was cultured in nutrient broth (Hi-media) at 37°C under shaking conditions (200 rpm). The list of microorganisms used in the study is shown in Table 1.

**TABLE 1A:** Strains used for the study.

Sl. No.	Indicator organisms	MTCC No.
1	*Pseudomonas putida* (−ve)	2492
2	*Corynebacterium callunae* (+ve)	700
3	*Pseudomonas aeruginosa* (−ve)	1934
4	*Salmonella typhi* (−ve)	New isolate
5	*Enterococcus gallinarum* (+ve)	7049
6	*Streptococcus thermophilus* (+ve)	New isolate
7	*Staphylococcus aureus* (+ve)	1430
8	*Vibrio harveyi* (−ve)	7954
9	*Streptococcus mutans* (+ve)	497
10	*Vibrio cholerae* (−ve)	3904
11	*Bacillus cereus* (+ve)	430
12	*List*eria monocytogenes (+ve)	839
13	*Clostridium perfringens*	450
14	*Lactobacillus plantarum*	Lab Isolate
15	*Enterococcus raffinosus*	Lab isolate

*−ve represents Gram-negative and +ve represents Gram-positive bacteria.*

### Isolation of BAMP Producer

More than a hundred bacterial strains were isolated from the Himachal Pradesh Traditional Fermented Foods such as mango pickle, rice-based fermented foods, and bamboo shoots. The source material was dissolved (approximately 10 g/ml) in peptone water, and appropriate serial dilutions were made, spread to get an isolated colony. Subsequently, microorganisms such as *Salmonella* spp., *Listeria* spp., and *Vibrio* spp. were screened for their antimicrobial activity against common food spoilage. The isolated colonies were inoculated and grown in MRS, LB, BHI, and NB from 12 to 18 h. The cells were harvested by centrifugation (9,000 × *g* for 15 min at 4.0°C). The cell-free supernatant and/or crude extract of 15 isolates were subjected to well-diffusion assay with various food spoilage microorganisms as indicators.

### Identification and Characterization of the Isolate

The isolated microorganism was grown in a nutrient medium, and genomic DNA was isolated using Gene JET Genomic DNA Purification Kit (Thermo Fisher Scientific). The genomic DNA was used for 16S rRNA-based identification. The universal forward and reverse primers were used for polymerase chain reaction (PCR) amplification of 16S rDNA, which were 27F/1492R (5′-AGAGTTTGATCCTGGCTCAG-3′ and 1492R, 5′-GGTTACCTTGTTACGACTT-3′) with *Taq DNA polymerase* (Thermo Scientific DyNAzyme II DNA Polymerase). Thermal cycling parameters followed are as follows: hot-start at 95°C for 2–4 min, followed by 30 cycles of denaturing at 94°C for 40 s, annealing at 50°C for 40 s, and extension at 72°C for 1 min. Final extension for 5 min at 72°C. Subsequently, the PCR product was subjected to 1.0% agarose gel, sequenced, and submitted to NCBI. DNA gyrase-based phylogenetic studies were also followed for taxonomic characterization (data not shown).

### Growth Curve Studies of the Producer Strain

In all the studies, a single isolated colony was obtained by streaking cultures from the glycerol stock. The streaked agar plate was incubated for 12–16 h, at 37°C. The single isolated colony of *B. paralicheniformis* was inoculated in nutrient broth, incubated for 12 h at 37°C at 200 rpm shaking condition. Subsequently, the overnight culture was subcultured to obtain an optical density (OD) of 0.1 at 600 nm. The resulting fresh culture was grown until it reached the stationary phase. During the growth, the OD of the culture was measured at 600 nm after every hour using an ultraviolet (UV) spectrophotometer (Shimadzu analytical PVT LTD, Mumbai, India). The growth curve was expressed in a graph by plotting OD against time. As reported by Stevenson and Aalto-Araneda, we followed the measurement of OD as it is deemed suitable for the relative comparison of growth patterns of microorganisms than absorbance (Stevenson et al., 2016; Aalto-Araneda et al., 2020).

### Antimicrobial Peptide Production Kinetics

*B. paralicheniformis* was grown in nutrient broth at 37°C under shaking conditions as explained above. The production kinetics of the antimicrobial compound was estimated by collecting the culture supernatant after every hour of growth for 12–16 h. The resulting supernatant (1.0 ml) was centrifuged at 9,000 × *g* for 20 min at 4.0°C which was later filtered using 0.22 μm sterilized cellulose membrane (Millipore). The supernatant was used for preliminary studies, later subjected to agar well-diffusion assay against the indicator strain *Vibrio harveyi*. The zone of inhibition (ZOI) (activity) was measured using an antibiotic zone measurement scale in millimeters (mm).

### Well-Diffusion Assay With Different Indicator Strains

The cell-free supernatant produced as explained above was used for Agar well-diffusion assay (Choyam et al., 2015) to detect the antimicrobial activity. The cell-free supernatant (CFS) was centrifuged at 9,000 × *g* for 30 min at 4.0°C, and the supernatant was passed through a 0.22-μm membrane filter (vacuum-driven membrane filter, Hi-media), followed by ammonium sulfate precipitation, and dialyzed. The Gram-negative and Gram-positive pathogenic and food-spoilage microorganisms were used as indicators, as listed in Table 1. The culture of the indicator strain is grown overnight and mixed with BHI soft agar (0.75%), then subsequently poured over the previously prepared BHI agar and allowed to solidify. The wells (approximately 0.6 mm diameter) were made in the agar plate using a sterile borer. The filtered supernatant (30 μl) was added to the well and incubated for 12 h at 37°C. Nisin was used as a positive control (based on the indicator). The ZOI was measured after 12 h of incubation using an antibiotic zone measurement scale. MRS/NB agar with 0.1% calcium carbonate (CaCO_3_) (neutralizes acidity caused by organic acids) was used as a control for well-diffusion assay (Sigma-Aldrich, Bengaluru, India).

### Overlay Assay

The chromatography-purified BAMP was subjected to tricine-SDS-PAGE. The gel was made in two parts, one part of the gel contained the molecular weight marker (lane 1) and purified protein (lane 2). The other part of the gel was subjected to fixation, later washed with 100 mM sodium phosphate buffer. Subsequently, SDS was removed by washing thrice with 2.5% Triton-X 100 for 3 h. Finally, the gel was washed with Milli-Q water to remove Triton X-100. The resulting gel was overlayed onto the Petri plates seeded with *V. harveyi*, which were incubated at 37°C overnight.

### Percent Viability of the BAMP-Treated *V. harveyi*

The overnight grown culture of the indicator microorganism (*V. harveyi*) was subcultured (1.0%) in a fresh MRS broth. The experiment was divided into four groups. One group was maintained as a control without the addition of any antimicrobials. BAMP CFS (crude) and BAMP powder were added to the second and third groups, respectively, soon after the subculture in a volume and quantities as per the requirement. The culture samples were collected at regular intervals (1 h each) for 12 h (for viability studies). They were serially diluted and plated to determine the colony-forming units (CFU/ml). The results were compared with negative and positive controls.

### Effect of pH, Temperature, Different Proteolytic Enzymes, Food Preservatives, and Food-Grade Metal Salts

The BAMP protein solution was adjusted at pH 1.0–14.0 using 1.0 N HCl and 1.0 N NaOH and incubated at 37°C for 2 h. The activity against the indicator microorganism (*V. harveyi*) was checked by approximately neutralizing the pH to 8.0. To understand the effect of temperature, aliquots of the culture supernatant were taken and treated at different temperatures, including 80°C for 1 h and 100°C for 30 min, and were then autoclaved for 20 min. The untreated protein solution was used as a control.

The sensitivity of the antibacterial substance (BAMP) to various physiological enzymes was evaluated. Aliquots of the protein solution (CFS) at different pH values were incubated (1:1, *v*/*v*) with an enzyme (1.0 mg/ml) and their respective controls for 2 h at 37°C. Similar experiments were conducted with sodium dodecyl sulfate (SDS), Tween 20, Tween 80, and Triton X-100, which were incubated with the antimicrobial compound (0.1 mg/ml) at a final concentration of 1.0% (*v*/*v*) for 5 h at 37°C. The antimicrobial activity of BAMP was checked against the indicator microorganism, *V. harveyi*. The permitted concentrations of the detergents alone were tested for their antagonistic activity, and they were taken as –ve controls. All the experiments were conducted thrice, and the average was considered for theoretical performance.

To understand the effect of various metal salts (used as preservatives) on antagonistic activity, BAMP was incubated with various food-grade metal salts such as MgSO_4_, FeSO_4_, MnCl_2_, AgNO_3_, ZnSO_4_, CdCl_2_, CuSO_4_, and CaCl_2_ at a final concentration of 1.0 mg/ml for 1 h at 37°C, and the activity was measured against the indicator microorganism. Untreated samples and metal salts at their final concentration were taken as controls (Kumar et al., 2015).

Finally, the effect of various food preservatives and their effect on antimicrobial activity of BAMP was followed (as above) by considering sodium chloride, sucrose, acetic acid, ascorbic acid, benzoic acid, sodium benzoate, and sodium sulfite. Here, BAMP was mixed with different concentrations of preservatives, and the activity checked against the most common food pathogens (*L. monocytogenes*, *V. harveyi*, *S. mutans*, and *S. aureus*). The ZOI around each well was measured using an antibiotic zone measuring scale. The residual activity and percentage activity reduction of BAMP upon different treatments were calculated, tabulated, and plotted separately (all the experiments were performed thrice).

### Purification of the Antimicrobial Agent

The culture of *B. paralicheniformis*, grown overnight, was subcultured in 1 L of nutrient broth and incubated for 72 h at 37°C under shaking conditions (200 rpm). The culture was harvested by centrifugation at 9,000 × *g* (radius of rotor/diameter 100 mm) for 30 min at 4.0°C. The supernatant was filtered through a 0.22-μm filter. The filtered supernatant was subjected to 0–70% ammonium sulfate precipitation and dialyzed. The dialyzed sample was diluted with NaCl and phosphate buffer and pH adjusted to 7.0. Before, the gel filtration (Superdex 75, Sigma Aldrich) column was equilibrated with (one column volume) 50 mM phosphate buffer and 150 mM NaCl, pH 7.0 (Amersham Biosciences, FPLC, Uppsala, Sweden). Subsequently, the sample (2.5 ml) was loaded and its flow through was tested for the activity. Later, the column was washed with one column volume of wash buffer. Suitable flow rate (1.0 ml/min, 76.4 cm/h) such as the long column with a slow flow rate was considered as it may give good resolution. Finally, the protein was eluted with 150 mM phosphate buffer and 2.0 ml fractions were collected. The whole procedure of purification was followed for 130 min. Fraction collection was initiated at 40 min and terminated at fraction number 40. Each fraction was subjected to well-diffusion assay/antimicrobial activity.

### Determination of Minimum Inhibitory Concentration of Purified BAMP

The minimum inhibitory concentration (MIC) of the purified antimicrobial peptide/protein (AMP) was (against Gram-negative and Gram-positive microorganisms, antagonistic activity against probiotic microorganism) determined using the microbroth dilution method (methods for antimicrobial dilution and disk susceptibility testing of infrequently isolated or fastidious bacteria) (Eucast). [Determination of minimum inhibitory concentrations (MICs) of antibacterial agents by broth dilution, 2003; Temitope et al., 2020]. The method is as follows; *V. harveyi* test strain was grown in MRS at 37°C at 180 rpm. The culture was grown until the mid-exponential growth phase with an OD_600_ of 0.4. Subsequently, the culture was adjusted to the final bacterial count of ∼4 × 105 CFU/ml. A hundred microliter of sterile media containing the twofold serial dilutions of the purified BAMP was added to each well. Then, 100 μl of prepared culture was added and the plate was incubated at 37°C for 18 h. The lowest concentration with no visible growth in the well was considered the MIC.

### Hemolytic Assay

The method of hemolysis by Rodríguez et al. (2014) was followed. The method is as follows: human blood (5.0 ml) was collected and centrifuged and the pelleted erythrocytes were washed thrice with phosphate-buffered saline (PBS, 150 mM NaCl at pH 7.0); 20 % (*v*/*v*) of erythrocyte suspension was made in the PBS solution. Different concentrations of purified BAMP 2.5, 5, 10, 50, and 100 μg/ml were incubated with 1:5 dilutions of 20% (*v*/*v*) of RBC at 37°C for an hour. Later, the sample was centrifuged and the OD_450__nm_ was estimated. The positive control for the study was 1.0% (*v*/*v*) Tween 20 in PBS solution buffer alone. The following equation was used to calculate the percentage of hemolysis.

(A450 of the peptide-treated sample – A450 of the buffer-treated sample) × 100/(A450 of the Tween 20-treated sample – A450 of the buffer-treated sample).

### Mass Spectra Studies of BAMP

Following gel filtration chromatography, the homogenous and purified BAMP was subjected to nano-LC-ESI-MS (Agilent 6550 I funnel QTOF).

### Sample Preparation

Purified protein sample at 25.0 μm in 50 mM sodium phosphate buffer was subjected to zip tip with 0.6 μl of C18 resin (Merck Millipore, India). First, the column was regenerated with 10 μl of acetonitrile (MS-MS grade). Followed by equilibration with 0.1% trifluoroacetic acid (TFA) at 10 μl × 10 times. The protein sample was loaded, subsequently, washed with 0.1% TFA and finally eluted with 50 and 80% acetonitrile (ACN). The samples were speedVac dried, and the resulting pellet was resuspended in MS-grade water.

### Mass Spectrometric Analysis of Protein

MS conditions are as follows: nitrogen as curtain gas, ISVF 5,500 V at 100°C, DP 100, and Tof range 100–2,000 *m*/*z* ion source. The selected multiple-charged ions were subjected to MS/MS analysis using collision energy at 30–45. MS data were acquired using a data-dependent top10 method dynamically choosing the most abundant precursor ions from the survey scan.

### Scanning Electron Microscope Analysis of the Indicator Strain *V. harveyi* After BAMP Treatment

The culture of the indicator strain (*V. harveyi*) grown overnight was subcultured to obtain 0.01 OD_600_ and mixed with the purified antimicrobial compound (0.01 mg/ml). The mixture was incubated for 12 h at 37°C under shaking conditions (200 rpm). One-milliliter sample was centrifuged at 9,000 × *g* for 10 min to pelletize the cells and fixed in 2.0% glutaraldehyde solution for 12 h. Subsequently, they were subjected to a series of alcohol washes (10–100%) and analyzed under scanning electron microscopy (SEM). The surface images obtained were compared with the control. The experiments were performed in triplicate, along with the control (untreated).

### The Bacteriostatic Effect of BAMP on Fluorescent-Labeled *S. typhi*

The indicator strain, *S. typhi*, was labeled with a green fluorescent protein (GFP) by transforming the pFU95 GFP plasmid (a kind gift of Dr. Tuli, IMT, Chandigarh). The labeled cells cultured overnight in LB–ampicillin broth. Then, the subcultured samples were allowed to reach 0.5 OD, after which BAMP was added. A *Salmonella* GFP without BAMP treatment was maintained as control, and samples were drawn at regular intervals and processed through fluorescence microscopy. The results were compared with the untreated control.

### Impact of BAMP on Ground Chicken Meat and Reduced CFU

Fresh chicken meat (boneless) from the local market was minced using a meat mincer (sterile). The resulting 10 g of meat slurry (in 10 ml of phosphate buffer) was taken, and the experiment was divided into two batches representing two different temperatures 37°C and 4.0°C for 36 h and 16 days, respectively. One group was maintained as a fresh group, without the inoculation of the pathogenic strain. The other groups were mixed with 1.0% inoculum (0.5 OD) of *L. monocytogenes*, subdivided into three groups. The three subgroups were BAMP treated (3.0 μg/ml) and untreated (negative control). The volume and quantities of antimicrobials taken correspond to their MIC (3.0 μg/ml). An aliquot (representing) of 0.1 g from 10 g of each group was taken on days 1, 4, 7, 10, 13, and 16 from samples stored at 4.0°C and 12, 24, and 36 h from those incubated at 37°C. The *L. monocytogenes* CFUs were estimated from a serial dilution of each aliquot by plating. The results were compared with positive and negative controls.

### Whole-Genome Sequencing, *de novo* Genome Assembly, Gene Prediction, and Functional Annotation

Genomic DNA was isolated from the *B. paralicheniformis* using the Qiagen gDNA extraction kit (Qiagen, Hilden, Germany), and the whole-genome sequencing was performed on the Illumina HiSeq 2,000 sequencing platform using a paired-end library, resulting in 150-base pair (bp) paired-end reads and an average coverage of 170-fold. Raw paired-end reads were evaluated, and quality control was carried out using FastQC^1^ and Trimmomatic (Bolger et al., 2014) (parameters: ILLUMINACLIP: 2:30:10, LEADING: 3, TRAILING: 3, SLIDING WINDOW: 10:30, MINLEN: 100) to obtain a set of clean paired-end reads. After preprocessing, FastQC was again used to report features of the preprocessing libraries and to verify the effectiveness of read trimming. After filtering, short reads were assembled using the SPAdes Genome Assembler with default parameters (Bankevich et al., 2014).

Prodigal (version 2.6.2) (Hyatt et al., 2010) was used for gene prediction in the *Bacillus* spp. sample draft genome, while BAGEL-4 (Medema et al., 2011) and anti-SMASH (van Heel et al., 2018) were used to predict the biosynthetic gene clusters for secondary metabolites and antimicrobial peptides with default parameters. The whole-genome sequence of *B. paralicheniformis* was submitted in NCBI, and the accession number is WHJA00000000. The above process followed may predict the presence of possible antimicrobials/bacteriocins in the genome.

## Results and Discussion

### Identification of the Producer Strain Using 16S rDNA and Gyrase A Phylogenetic Analysis

The amplified full-length (1,500 bp) 16S rDNA and the resulting DNA sequences were analyzed by subjecting them to NCBI BLAST. BLAST results above 99.8% were identified as *B. paralicheniformis*. The 16S rRNA sequence was submitted to the NCBI sequence submission portal, and the accession number is MG183675. Table 1 contains all the strains used in the study. For further confirmation of *Bacillus* speciation, phylogenetic analysis and taxonomic studies of 16S rRNA and gyrase A (Gyr) A-conserved sequences were followed (data not shown). The whole-genome sequence of the producer strain was submitted in the NCBI WGS portal (accession No. WHJA00000000).

### Growth Curve of the Producer Strain

**FIGURE 1:**
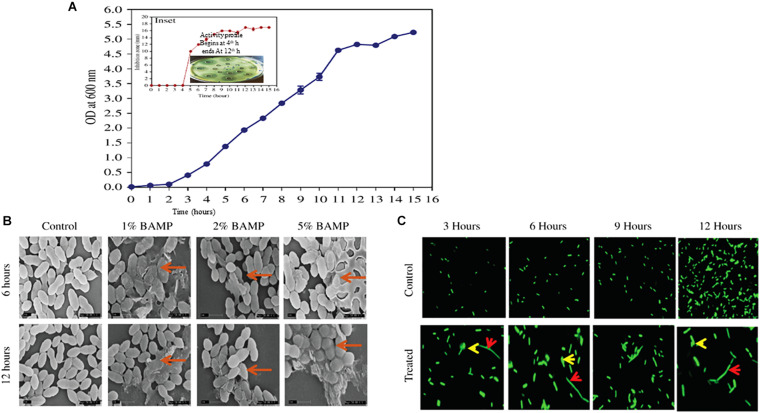
**(A)** Growth curve of *B. paralicheniformis*. Inset shows antimicrobial protein production kinetics. The indicator strain used is *V. harveyi*. The inset shows diffusion assay of the zone formation which begins after 4 h of growth, and the peak ZOI continued to 12 h. **(B)** SEM images of the different percentages of BAMP-treated *V. harveyi* cells in its 6 and 12 h of growth. The arrows indicate the swollen and damaged cells. **(C)** Effect of BAMP on florescent-labeled *S. typhi* at regular intervals of growth is shown in comparison with the control. The elongated bacteria are shown with a red arrow; bulged cells are indicated with a yellow arrow.

The growth curve of *B. paralicheniformis* is shown as OD_600_ for the culture against time (Figure 1A). The growth curve studies were conducted as per the materials and methods, and the growth curve was observed to be sigmoidal. However, a few unusual observations were made wherein as soon as the growth reaches the stationary phase, the formation of huge amounts of mucus-like material was observed. Furthermore, the mucous formation was observed particularly when the bacteria were grown in the static condition and for long periods above the stationary phase. The harvesting of the cells becomes difficult once mucous formation sets in. However, the mucoid material formation does not negatively affect either inhibition or prevention of the production of bacitracin. The presence of residual mucus affects obtaining pure cell-free supernatant and a major problem in downstream processing occurs.

### Antimicrobial Production Kinetics

Growth and activity kinetics are shown in Figure 1A and inset, respectively, which demonstrate bacitracin synthesis and production set within 5 h of growth (inset). The secretion is rapid as it does not stop and continues even in a stationary phase. Therefore, the minimum growth of 5 h may be sufficient to synthesize the bacitracin. After 8–10 h of growth, harvesting is carried out to isolate the bacitracin. Inset plate shows the well assay of each bacterial supernatant collected at different time points and also the beginning of the secretion of BAMP and the end of secretion of BAMP. The collected CFS was tested through a well-diffusion assay by using various Gram-positive and Gram-negative microorganisms as indicators. The results are enclosed in Table 1B.

**TABLE 1B:** Inhibitory activity of BAMP against Gram-positive and Gram-negative indicator species.

Sl. No.	Indicator organisms	Zone of inhibition of BAMP (mm)
1	*Pseudomonas putida* (−ve)	10
2	*Corynebacterium callunae* (+ve)	12
3	*Pseudomonas aeruginosa* (−ve)	12
4	*Salmonella typhi* (−ve)	10
5	*Enterococcus gallinarum* (+ve)	15
6	*Streptococcus thermophilus* (+ve)	10
7	*Staphylococcus aureus* (+ve)	11
8	*Vibrio harveyi* (−ve)	13
9	*Streptococcus mutans* (+ve)	14
10	*Vibrio cholerae* (−ve)	10
11	*Bacillus cereus* (+ve)	11
12	*List*eria monocytogenes (+ve)	16
13	*Clostridium perfringens*	32
14	*Lactobacillus plantarum*	NA
15	*Enterococcus raffinosus*	10

### Scanning Electron Microscope Analysis

The BAMP-treated cells showed morphological changes compared with the control. The cell samples treated with BAMP were found to be swollen and elongated; these may either be due to the inhibition of cell division or inhibition of separation of daughter cells after cell division. Cell bursting and leakage of the cytoplasmic contents were observed in the treated cells. The nature of morphological changes upon treatment with different percentages of BAMP is shown in Figure 1B. The activity of BAMP increases as the percentage of BAMP in the reaction increases. The highest BAMP activity was observed at the 5.0% level, where cell wall disruption and lysis, the presence of swollen cells, and cell debris may be observed (marked by the red arrow in Figure 1B). The PI-labeling studies show bright-colored diffused cells, indicating cell lysis by BAMP (data not shown). Cell lysis begins at 6 h of 1% BAMP exposure; as the concentration of BAMP increased from 1 to 5%, the incidence of lysis increased. The severe cases of cell lysis and aggregation were observed when the cells were incubated for 12 h with larger quantities of BAMP.

### The Bacteriostatic Effect of BAMP on Fluorescent-Labeled *S. typhi*

The fluorescence microscopic images of the BAMP-treated cells showed it interfered with division and resulted in elongated cells when compared with the untreated control at regular intervals during growth. Two different kinds of cells were observed in lysed cells: elongated cells and diffused cells. This indicates a compromised cell wall and cell lysis. This shows the bacteriostatic effect of BAMP on *S. typhi* cells (Figure 1C). Here, we could also see elongated, abnormal GFP-expressing cells. A few cells were ruptured due to BAMP action where the GFP is observed coming out of the cells (arrows in the figure indicates rupture). There is an overall decrease in the GFP levels after BAMP treatment in comparison with the control *Salmonella* GFP (Figure 1C, 12 h sample).

### Effect of pH and Temperature

BAMP was subjected to different temperatures ranging from 4.0 to 121°C (autoclaving) for 20–60 min. The results show no change in the activity of BAMP. At 4.0–80°C, exposure does not reduce the activity, but upon exposure to 100°C, a loss of 10% activity was observed, which was further reduced to 20% at 121°C. This shows that BAMP is thermostable. To understand the effect of pH (1.0–13.0), BAMP was treated with different pH values. No drastic changes in BAMP activity and a very marginal reduction in the activity at pH 2.0 and 9.0 (the reduction was 20%) were observed. However, at pH 11.0 and higher, the activity was negligible. The purified antimicrobial peptide showed good activity. The temperature and pH do not negatively affect BAMP.

### Effect of Physiological Enzymes on the Activity of BAMP

Even after treatment with physiological enzymes, including chymotrypsin, trypsin, pepsin, protease, proteinase K, and catalase for 5 h at 37°C, the antimicrobial compound continuously maintained its activity (Table 2A). Table 2A shows the residual activity of BAMP after treatment with physiological enzymes. Hyperactivity of BAMP was observed (83%) in the presence of proteinase K (ZOI 10.5 mm). The ZOI of the BAMP treated with physiological enzymes ranges from 10.5 to 12.0 mm. This is another factor that makes BAMP suitable for food industries that process hydrolysates and protein concentrates since it can be degradable.

**TABLE 2A:** Effect of physiological enzymes on the activity of BAMP.

ENZYME	BAMP ZOI (mm)
Control	13.0
Chymotrypsin	11.5
Trypsin	12.0
Pepsin	10.5
Protease	10.5
Proteinase K	10.5
Catalase	10.5

The percent residual activity of the physiological enzyme, surfactants, and metal salt-treated BAMP shows that there is a loss of 20% activity. The ZOI of the BAMP treated with various surfactants and chloroform ranges from 10.5 to 12.5 mm. Therefore, a few chemical food preservatives do not negatively affect their function (Table 2B).

**TABLE 2B:** Effect of surfactants and chloroform on the activity of BAMP.

Treatment	BAMP ZOI (mm)
Control	13.0
SDS	12.5
Tween 20	10.5
Tween 80	10.5
Triton x-100	12.5
Chloroform	11.5

### BAMP Stability Test in the Presence of Various Detergents/Effect of Surfactants and Chloroform on the Activity

As per the rules of the Food Safety and Standards Authority of India (FSSAI), permitted amounts (of surfactants and chloroform in the food industry) were used for these studies. The antimicrobial compound maintained its activity in the presence of surfactants such as SDS, and Triton X-100 nominal loss of 10–20% activity was observed with the other surfactants (Table 2B). This further implies that BAMP is an efficient food preservative as well as an antimicrobial compound. The residual activity and the reduction of activity of BAMP treated with various detergents are shown in Table 2B.

### Effect of Food-Grade Metal Salts

As per the regulations of the FSSAI and the United States Food and Drug Administration (USFDA), various metals were considered to understand their effect on the antagonistic activity of BAMP. Similarly, a few metals do negatively affect BAMP, e.g., CuSO_4_ drastically inhibits its activity (no activity). However, BAMP is more effective in the presence of most of the metals. The BAMP activity was unaffected when subjected to seven different metals out of eight (Table 2C). The residual and percent reduction of the activity of the BAMP after treatment with food-grade metal salts are presented in Table 2C. The ZOI of the BAMP exposed to food-grade metal salts is not affected drastically wherein the ZOI in the case of BAMP ranges from 0 to 12.5 mm. Here, BAMP shows a superior function. The effect of BAMP on another infectious pathogen *V. harveyi* was evidenced through viability studies (data not shown). It is suggested not to use BAMP in food material that contains CuSO_4_.

**TABLE 2C:** Effect of food-grade metal salts on the activity of BAMP.

Metal salts	BAMP ZOI (mm)
Control	13.0
MgSO_4_	10.5
FeSO_4_	10.5
MnCl_2_	10.5
AgNO_3_	12.5
ZnCl_2_	11.5
CdCl_2_	13.5
CuSO_4_	0
CaCl_2_	10.5

*The values are the results of three independent experiments.*

*ZOI, Zone of Inhibition.*

### Effect of Food Preservatives on the BAMP Activity

As per the prescription by the FSSAI/USFDA, various food preservatives were considered for the study. The results showed that the antimicrobial compound maintained its activity in the presence of preservatives. Virtually, the food preservatives do not inhibit the action of either BAMP. Tables 2A–C shows 100% residual activity, taken into consideration during the final calculations of activity reduction. This value gives actual activity only.

### Purification of BAMP

**FIGURE 2:**
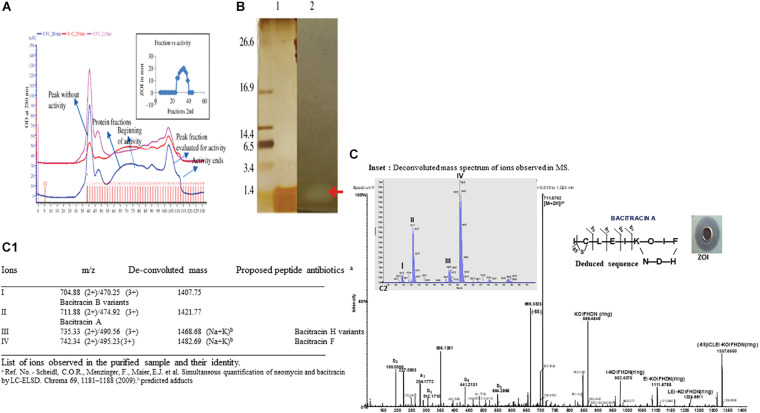
**(A)** Elution profile and activity profile of the BAMP-purified fraction. The antimicrobial activity of the fractions is showed in the inset. **(B)** Tricine-SDS-PAGE of purified BAMP. Lane 1: marker; lane 2: purified BAMP; lane 3: direct overlay of tricine-SDS-PAGE gel demonstrating ZOI indicating the activity of purified BAMP. **(C)** MS-MS spectrum of purified BAMP (inset) showing the well-diffusion assay/ZOI. (a) LC-MS profile of active fraction of antimicrobial compound of BAMP. Assignment of b and y ions. Amino acid sequence deduced from the MSMS data. The inset shows the deconvoluted mass spectrum of peaks I, II, III, and IV and were observed and demarcated. **(C1)** The List of ions observed in the purified sample and their identity.

The ammonium sulfate-precipitated protein after dialysis was subjected to a Superdex 75 gel filtration column. Figure 2A shows the elution profile of BAMP. We can see that the peak is not steep but is very shallow; it shows a zigzag manner, and elution begins after 35 min and proceeds to 130 min. Almost all the fractions were subjected to Bradford analysis, and activity was observed only in the fractions that eluted after 90 min and continued for 115 min. The peak fractions could be observed from 25 to 40 min. The eluted peaks were collected and the activity checked (by a well-diffusion assay). The elution profile and activity profile (inset) of the purified fractions were plotted (Figure 2A, inset). The activity of gel filtration fractions begins with fraction numbers 28–31 in low activity at around 7.0–8.0 mm ZOI. The highest activity was observed between the fraction numbers 32 and 39. Here, the ZOI ranges from 10.0 to 12.0 mm. Therefore, the elution peak with activity is between 28 and 40 fractions of 2.0 ml each. The ZOI was seen from the fraction number 33 onwards and continuing to 39. It begins at the 95th minute and ends at the 115th minute of elution profile, as shown in Figure 2A. Figure 2A (inset) shows the beginning and the end of the active fractions with time.

BAMP purification is shown in Figure 2A (inset). Table 3A shows the purification table of BAMP. The homogenous purified and electrophoretically separated single protein band is shown in Figure 2B (lane 1). Subsequently, the purified protein was subjected to an in-gel activity assay—zymogram and shown in Figure 2B (lane 2). The gel, as well as the zymogram, shows an approximate molecular weight of 1.4 kDa. The BAMP CFS and BAMP powder were subjected to check their effective concentrations against various food contaminants. The results show that BAMP does not work against *Pseudomonas putida* but is antagonistic to *Pseudomonas aeruginosa*. The results also indicate that BAMP MIC ranging from 2.4 to 6.0 μg (Table 3B) varies with different indicator strains. The same protein also tested their ability to kill probiotic microorganisms such as *Lactobacillus plantarum* and *Enterococcus* spp. It was understood that BAMP (Table 3C) is not antagonistic to *Lactobacillus* and functional against *Enterococcus* spp. The specific target function of BAMP renders it suitable for human therapy and eubiosis. The eubiosis nature of BAMP further supports its suitability as a therapeutic protein in the maintenance of gut microbiota and elimination of pathogenic and disease-causing microorganisms.

**TABLE 3A:** BAMP purification table.

Purification stage	Volume (ml)	Total protein (mg)	Total activity (AU)	Specific activity (AU/mg)	Purification (fold)	Yield (%)
Crude supernatant	1,000	100	33,320	333.2	1	100
Ammonium sulfate precipitation	20	23.6	10,662	451.77	1.335	31
Superdex-75 gel filtration	16	0.265	2,665	10039.9	30	7

**TABLE 3B:** MIC of BAMP supernatant and spray dried powder (concentration in micrograms).

Sl. No.	Indicator strain	BAMP supernatant (μg)	BAMP powder (μg)
1	*Enterococcus gallinarum*	3.0	6.0
2	*Corynebacterium callunae*	2.4	3.0
3	*Listeria monocytogenes*	2.4	3.0
4	*Streptococcus mutans*	2.4	6.0
5	*Staphylococcus aureus*	6.0	12
6	*Micrococcus luteus*	2.4	3.0
7	*E. coli*	3.6	6.0
8	*Vibrio harveyi*	3.0	1.2
9	*Pseudomonas putida*	–	–
10	*Pseudomonas aeruginosa*	–	12
11	*Streptococcus thermophilus*	Bacteriostatic	–
12	*Salmonella typhi*	Bacteriostatic	–
13	*Vibrio cholerae*	Bacteriostatic	–

**TABLE 3C:** The activity of the purified antimicrobial compound against probiotic microbes. The activity was expressed as a zone of inhibition.

Probiotic strain	BAMP
*L. plantarum*	NA
*E. raffinosus*	10 mm

*NA, No Activity.*

### The Minimum Inhibitory Concentration of BAMP

The MIC was observed to change based on the protein condition, and the crude supernatant MIC was 3.0 μg/ml, ammonium sulfate-precipitated protein MIC was 2.1 μg/ml, and MIC of the purified antimicrobial compound was 0.124 μg/ml. The MIC was determined using the microbroth dilution assay. The MIC was found to be 3.0 μg/ml against the indicator strain *V. harveyi*. Various indicator strains were used to find a suitable strain, and it was found that *V. harveyi* is the best indicator and consistent results were obtained. Most importantly, it is pathogenic, contagious, ubiquitous, and food contaminating. *Vibrio* spp. is one of the major contaminants in the dairy and aquatic system. We also used *Salmonella* as this is also one of the food contaminants. Outbreaks of *V. harveyi* are known to be very common as stated by Zhang et al. (2020). Subsequently, during the year 2020, Ogunbanwo et al. (2003) reported milk contaminated with *V. harveyi*. It is a major contaminant of dairy foods as well as the aquatic system; therefore, we chose *V. harveyi* for the study. MIC determined is shown in Table 3B.

### Mass Spectra/MS Analysis

The MS results of the purified BAMP showed the presence of a 1.4-kDa peptide (Figures 2A,B). Tricine-SDS-PAGE of the purified BAMP showed a protein band at the range of 1.4 kDa (Figure 2B, lane 1). The corresponding zymogram indicates the activity of the peptide (Figure 2B, lane 2). The LCMS data was processed using proteome discoverer software against UniProt *Bacillus* database at MS1 and MS2 tolerance of 10 ppm and 0.5 Da, respectively (Figures 2C,C1). The identified peptide is bacitracin as reported earlier (Singh et al., 2019).

MS analysis of peptide sample indicates the presence of majorly triply and doubly charged ions at *m*/*z* 470.25 (3+), 474.92 (3+), 495.23 (3+), 711.88 (2+), 735.33 (2+), and 742.34 (2+). The deconvolution of the spectrum revealed the ions with mass—1,407.75, 1,421.77, 1,468.68, and 1,482.69 Da (Figure 2C, inset a, C1), corroborating to the mass of the peptide determined through SDS-PAGE. Furthermore, the evaluation of 711.88 ion tandem spectra showed the presence of fragment ions that could match bacitracin A (Figures 2C,C1; Govaerts et al., 2003; Landeen et al., 2016). In the case of ions at 1,468.68 and 1,482.69 Da, we observed 61 Da increment compared with 1,407.75 and 1,421.77, respectively, that could correspond to Na^+^K adduct of keto-thiazole form. The list of identified bacitracin variants is given in Figure 2C1. Mass spectrum data indicate that there are four different kinds of peptide antibiotics/bacitracin antimicrobials as bacitracin A variants, bacitracin B variants, bacitracin F variants, and bacitracin H variants.

The compound was further analyzed through tandem MS and amino acid analysis, and the presence of isoleucine, cysteine, leucine, aspartic acid, glutamic acid, isoleucine, lysine, ornithine, isoleucine, phenylalanine, histidine, aspartic, and arginine was found. We observed the presence of one non-standard amino acid ornithine (Figure 2C). Subsequently, it was observed that the MS/MS data also supported the amino acid composition.

We studied the MS/MS data extensively and annotated all the b and y ions present in the raw spectrum (Figure 2C, deduced sequence, inset). Lockhart and Abraham (1954) and Stone and Strominger (1971) stated that bacitracin A is a cyclic peptide containing 12 amino acid residues. For further confirmation of the presence of bacitracin at the genome level, we looked into WGS analysis focusing on the genes encoding for the synthesis of the bacitracin.

### The Effect of BAMP in Chicken Meat Contaminated With *Listeria*

**FIGURE 3:**
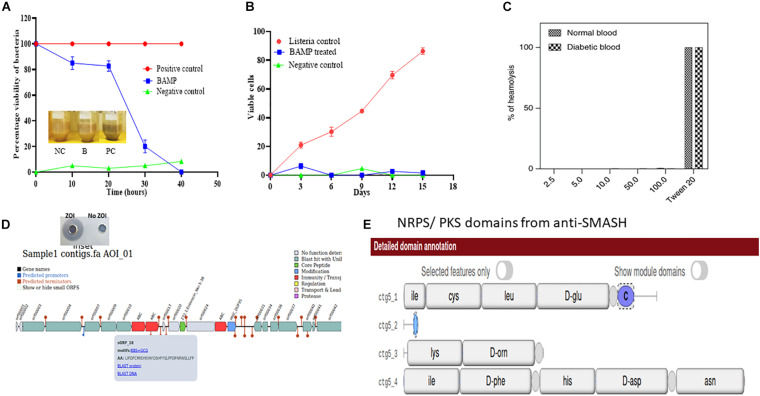
**(A)** Viability of *L. monocytogenes* in chicken meat on treatment with Nisin and BAMP for 36 h at 37°C and 16 days at 4°C. The picture in the inlet of graph A shows the differences in appearance of treated and untreated samples. NC, negative control; B, BAMP-treated samples; PC, positive control. **(B)** *L. monocytogenes* viability curves after treatment with BAMP. **(C)** Hemolytic activity of BAMP on healthy and diabetic human blood samples. **(D)** Antimicrobial biosynthetic gene cluster in the genome sequence of *B. paralicheniformis.* The inset shows ZOI and No-ZOI, predicted through BAGEL 3. **(E)** Non-ribosomal peptide predicted using anti-SMASH.

The percent viability of BAMP-treated *L. monocytogenes* was found to reduce (20% in 20 h) compared with the untreated *L. monocytogenes*. The BAMP-treated *L. monocytogenes* showed percentage viability similar to the negative control at 4.0°C. The viability of the cells gradually decreased at 37°C. The positive control turned blackish upon incubation, but the color of the treated sample was similar to the negative control. This shows the antimicrobial activity of samples against the indicator strain used (Figure 3A). BAMP shows 50% viability (50% dead) in 30 h (Figure 3B). Subsequently, around 10% viability was observed in 38 min, and 90% of *Listeria* was eliminated from the food at 37°C. A similar experiment at 4.0°C concluded an almost negligible number of viable cells. This further confirms the effect of BAMP and its use in the meat industry. The *Listeria* field trials envision that the use of BAMP does not change taste, color, and flavor. Without BAMP, the sample does not only lose its color but also its flavor and taste. This envisages BAMP as a potential biopreservation agent.

The wide activity range of BAMP against Gram-negative and Gram-positive microorganisms is shown in Table 1B. No activity of BAMP against probiotic microorganisms was detected (Table 3C). The activity of BAMP against *V. harveyi* was confirmed, and the viability percentage was analyzed using standard procedures (data not shown). The molecular characterization of BAMP such as tricine-SDS-PAGE, zymogram, and MS analysis predicted its molecular weight (Figures 2A–C). The BAMP was found internalized by the indicator strain through HPLC analysis, and the interaction of BAMP with DNA was confirmed by FTIR analysis (data not shown).

### Hemolytic Assay

As per the guidelines of FSSAI/FDA, to consider BAMP as a food additive/supplement, the protein should be non-hemolytic in nature. The hemolytic assay of the BAMP showed 0.43% hemolysis. The result based on the studies conducted to understand its hemolytic activity is negative (Figure 3C). This property makes the BAMP phenomenal, suitable as a food additive, as well as for therapy. As per the report, hemolysis values above 6 are non-phenomenal and not considered for therapy. The hemolytic attribute of BAMP strengthens its applicability to therapy.

### Whole-Genome Sequence Analysis and Prediction

Raw data sequences from Illumina HiSeq were assessed and filtered with FastQC and Trimmomatic to produce a high-quality set of reads. These whole-genome DNA samples yielded 4,374,835 raw paired-end reads with lengths of 150 bp. After the removal of adapters and low-quality data (quality scores ≤ 30), 2,256,832 clean reads (51.59% of the initial total) remained. The clean sequences were assembled into the draft genome of the *B. paralicheniformis* (accession No. WHJA00000000) in which 101 contigs were obtained with a total draft genome length of 4,200,259 bp (N50 of 287,402 bp and GC content of 45.86%). For functional analysis, we predicted 5,195 genes from the SPAdes-assembled contigs using Prodigal.

Moreover, we subjected the whole-genome sequence assembly to other tools like Antibiotics and Secondary Metabolites Analysis Shell (anti-SMASH) (Medema et al., 2011) and BAGEL-4 (van Heel et al., 2018) to discover the genes associated with the antimicrobials and secondary metabolites. Various bacteriocins were found in the WGS of *B. paralicheniformis* through BAGEL-4 and Anti-SMASH. Lichenisin and bacitracin are the two bacteriocins that are showing 100% similarity as per anti-SMASH data. According to anti-SMASH, bacitracin is localized in the contig number 6 and nucleotide position approximately from 15,000 to 45,000. Contig. Twenty-two comprise cystabiotic of 98 amino acids, whose sequence is identical to antimicrobial peptide LCI of *B. paralichemiformis.* Two bacitracins were observed at regions 2.2 and 22.2 and are NRPs with 100% similarity with the existing bacitracin. Figures 3D,E shows the gene arrangement in the genome sequence. The inset shows the well-diffusion assay of BAMP that was used for genome sequencing (zone of inhibition).

The bacitracin gene was also found in different species of *Bacillus* such as *B. licheniformis* ATCC 14580, *B. glycinifermentans*, even in *Paenibacillus* spp. and *B. paralicheniformis*. Therefore, the mass spectrum data coincide with the WGS data confirming the presence of a gene to the corresponding protein. Established along the gene arrangement shown in Figures 3D,E, it is noted that the bacitracin and its variants are arranged identically with other bacteriocins wherein it contains ABC transporters, hypothetical protein, and immunity protein in the same array. The genome sequence of the strain *B. paralicheniformis* has been deposited in the GenBank database, and Accession No. WHJA00000000 was obtained.

### BAMP Is a Potent Antimicrobial Peptide

The purified BAMP is thermostable. The activity could be seen after incubation at various temperatures ranging from 4.0 to 121°C. BAMP functions in a very wide range of pH from 2.0 to 9.0, where its activity is not affected, but above 9.0 in alkaline conditions, its activity is drastically reduced. Upon the treatment of BAMP with various surfactants such as SDS, Tween 20, Tween 80, Triton X-100, and chloroform, it was found that Tween 20 and 80 reduces the activity around 20% (Table 2B). Various food-grade metal salts and preservatives do not affect BAMP (Table 2C). BAMP does not create dysbiosis because it is harmless to the gut bacteria such as *Lactobacillus* and *Enterococcus* spp. (Table 3C). The compromised, broken, and aggregated *V. harveyi* after the treatment with the various concentrations of BAMP is shown in Figures 1B,C. This envisages the effect of BAMP causing cell damage. The case of *S. typhi* was an indication of the bacteriostatic effect of BAMP against certain indicator strains (Figure 1C). It was further confirmed that BAMP can permeabilize the cell membrane (Figure 1C). *Salmonella* with GFP clearly shows the compromised cell membranes. The percent viability of *V. harveyi* treated with BAMP shows that BAMP requires just 3–4 h for 50% lysis (Figures 3A,B). The chromatographic peak fractions shown in the inset were used for subsequent studies such as MS analysis. Homogenous and pure protein corresponding to approximately 1.4 kDa is shown in Figure 2B (lanes 1 and 2). Finally, the purified protein was subjected to MS analysis.

The virtual and real/field experimental setup studies conclude that BAMP controls the *Listeria* population in the meat (Figures 3A,B). This shows better efficiency of BAMP and confirms that BAMP could function in a food matrix as well. The purified BAMP is hemolytic negative as the present threshold considered for the therapy is much less and considered for therapeutic purposes (Figure 3C). Even after using 2.5–100 μg/ml, negligible activity was observed wherein Tween 20, a controlled detergent, showed the activity of 100%. BAMP is observed to have a character wherein it does not establish gut dysbiosis, establishing eubiosis by not harming the beneficial microorganisms such as *Lactobacillus*. The potent antimicrobial peptide BAMP was found to have many superior characteristics, making it desirable for the preservation of food from pathogens and an ideal therapeutic agent.

## Conclusion

The present work deals with the development of a potent next-generation antimicrobial agent that may be utilized as a food preservative. Although Nisin is extensively used as a preservative, there are a few features that limit its preservative functions, including its very limited antibacterial activity. Other limitations include its lack of function against very common food contaminants such as *S. typhi* and *S. thermophilus* that infect and spread rapidly in tropic regions.

In the end, we conclude that we successfully isolated, purified, and characterized a potential next-generation antimicrobial agent for the food industry. It is thermostable, physiologically enzyme resistant, and possesses wide spectrum activity. The molecule may be desirable for the various food industries, as it does not lose activity upon incorporation into the foods that contain metals, detergents, preservatives, and salts. The cloning, expression, and large-scale production/purification of the BAMP may help us to understand its therapeutic efficacy.

## Data Availability Statement

The datasets presented in this study can be found in the NCBI 16sr RNA and WGS repository under accession number WHJA00000000.

## Ethics Statement

The studies involving human participants were reviewed and approved by Institutional Biosafety Committee approval no. 012/IBSC/2020 reg. All methods were carried-out in accordance with relevant guidelines and regulations as well as a sentence confirming that informed consent was obtained from all subjects. The patients/participants provided their written informed consent to participate in this study.

## Author Contributions

RK: conceptualization, methodology, data curation, and writing – original draft preparation and reviewing and editing. SC: data generation and experimental work. PJ: involving in LC-MS studies. All authors contributed to the article and approved the submitted version.

## Conflict of Interest

The authors declare that the research was conducted in the absence of any commercial or financial relationships that could be construed as a potential conflict of interest.

## Publisher’s Note

All claims expressed in this article are solely those of the authors and do not necessarily represent those of their affiliated organizations, or those of the publisher, the editors and the reviewers. Any product that may be evaluated in this article, or claim that may be made by its manufacturer, is not guaranteed or endorsed by the publisher.

## Footnotes

1
http://www.bioinformatics.babraham.ac.uk/projects/fastqc/
